# Dark Tetrad personality traits and attitudes supportive of vindictive rape: mediating role of empathy

**DOI:** 10.1192/bjo.2025.10933

**Published:** 2025-12-22

**Authors:** Sara Veggi, Agata Benfante, Marialaura Di Tella, Henriette Bergstrøm, Franco Freilone, Lorys Castelli, Georgia Zara

**Affiliations:** Department of Law, University of Turin, Italy; Department of Psychology, University of Turin, Italy; School of Psychology, University of Derby, UK

**Keywords:** Dark Tetrad personalities, Dark Triad personalities, vindictive rape, empathy, myth

## Abstract

**Background:**

Research on gender-based violence has shown the influence of Dark Tetrad personality traits (i.e. Machiavellianism, narcissism, psychopathy and sadism) on the development and perpetuation of sexist attitudes and cognitions that justify or condone harmful behaviours.

**Aims:**

This study explored the potential mediating role of empathy in the relationship between the Dark Tetrad personality traits and support for vindictive rape, as a form of revenge for the perceived violation of traditional sexual norms.

**Method:**

A sample of 1548 adult individuals from the general community (67.3% female, age range 18–83 years) completed the Dark Triad Dirty Dozen, the Short Sadistic Impulse Scale, the Basic Empathy Scale and the Vindictive Rape Attitude Questionnaire.

**Results:**

The results showed that empathy partially mediated the relationship between sadism and attitudes supportive of vindictive rape, while a full mediation of empathy was found in the association between Machiavellianism, psychopathy and attitudes supportive of vindictive rape. Conversely, no significant association between empathy or vindictive rape and narcissism was observed.

**Conclusions:**

Empathy plays an important role in mitigating the effects of Dark Tetrad personality traits on support for vindictive rape. Given the global prevalence of violence against women, these findings are discussed in the context of a social climate that may reinforce the perpetuation of gender inequalities and sex-based stereotypes that are at the root of the acceptance of violence.

Violence against women poses an enduring social challenge, taking diverse forms and inflicting harm, often with long-lasting or lethal consequences.^1^ Sexually aggressive behaviour does not emerge in a vacuum but is deeply rooted in the surrounding social context. Perceptions of cultural norms, gender and sexual roles are therefore likely to play a role in defining interpersonal violence and the circumstances under which it can be considered ‘legitimate’. Cultural stereotypes are crucial in shaping expectations of acceptable behaviour for men and women.^2^ These stereotypes often ascribe inherent characteristics to women, labelling them as either ‘good’ (chaste, nurturing or pure) or ‘bad’ (promiscuous, hostile or seductive), and are closely linked to patriarchy-enhancing ideologies that disregard women’s rights.^3^ These evaluative logics operate within historical regimes of patriarchy that delimit women’s sexual and relational agency, producing normative ideals of modesty, passivity and docility as disciplinary techniques that regulate femininity.^4^ Simultaneously, heteronormative structures function as a matrix of intelligibility that naturalises heterosexuality and binary gender, assigning moral value to women’s sexual conduct and enacting social sanctions to deviations from prescribed norms.^5^ Through their mutual reinforcement, these systems consolidate gendered hierarchies that authorise men to surveil, discipline and punish women who do not conform to normative feminine sexual boundaries. Within this sociocultural and discursive field, patriarchal and heteronormative norms have historically inscribed women into positions of sexual restraint and relational subordination while positioning men as entitled subjects endowed with authority and sexual prerogative.^6^ These prescriptions operate as cultural scripts that both produce and constrain sexual subjectivities, stigmatising perceived departures from feminine respectability and sustaining relational power orders that legitimise men’s regulatory and punitive power over women’s choices.

Interestingly, studies suggest that personality plays a role in explaining differences in behavioural patterns and beliefs on interpersonal relationships and gender-based violence.^7^ Of specific interest in this context are the Dark Triad personality traits.^8^ The Dark Triad refers to a cluster of three personality traits (i.e. Machiavellianism, narcissism and psychopathy), which share characteristics related to manipulation, grandiosity, deceitfulness and callousness.^8^ Particularly, those who are high on Machiavellianism are especially likely to be emotionally detached and manipulative, while being high on narcissism is predominantly characterised by grandiosity and dominance. Finally, psychopathy is characterised by being high on insensitivity, cynicism and impulsivity. The Dark Triad has subsequently incorporated sadism (i.e. the pleasure arising from the physical or emotional suffering of others) in configuring what is referred to as the Dark Tetrad.^8^

Not surprisingly, these subclinical traits are considered salient in a range of antisocial behaviours, even in intimate settings.^9^ For example, individuals with high Dark Triad traits tend to display inappropriate reactions in interpersonal contexts.^10^ These reactions often include cold-heartedness, a diminished commitment to justice and less condemnation of deviant behaviour, which can undermine prosocial tendencies and lead to a reluctance to intervene in moral situations.^11^ Furthermore, when these traits intersect with a person’s sexual scripts (i.e. the expectations for how women and men interact in courtship and intimate relationships),^12^ they contribute to attitudes and beliefs that may facilitate sexual assault, such as victim-blaming and rape myth endorsement.^13,14^ In fact, the degree in which people believe in common rape myths has been found to be related to adherence to gender stereotypes about women.^15^ Such beliefs, albeit factually incorrect, are widely endorsed at multiple levels of society. Notably, this endorsement, which is particularly evident in men, is associated with lower empathy towards victims (i.e. the ability to infer, understand and share in the emotional experiences of others), which in turn has been consistently identified as a common characteristic of individuals with Dark Tetrad personality traits.^16^

Building on this evidence, recent research has increasingly focused on the role of reduced empathy as a psychological mechanism through which Dark personality traits translate into aggression and tolerance of sexual violence. Diminished empathic abilities have been empirically shown to link Dark Triad or Tetrad traits with increased aggression, victim-blaming and reduced moral inhibition: for example, research^16^ found that impaired cognitive and affective empathy partially mediated the relationship between psychopathy and indirect relational aggression. More specifically, in contexts of interpersonal and sexual violence, lower empathy has been found to promote endorsement of deviant sexual attitudes and diminish concern for victims, thereby functioning as a psychological mechanism connecting Dark personality traits to socially harmful behaviours. Recent work^17^ supports that empathy operates as a mediator of the effect of Dark personality traits on cyber- and interpersonal aggression. Some studies have found that individuals perceived as Machiavellians or psychopaths are more likely to be viewed as engaging in sexual harassment,^18^ especially by those who themselves exhibit higher levels of Machiavellianism and a proclivity for sexual harassment. Additionally, psychopathy has been linked to a greater likelihood of victim-blaming and a reduced tendency to hold perpetrators accountable in cases of sexual harassment.^14^ A more recent study^19^ also revealed a positive relationship between Machiavellianism and narcissism and the perceived responsibility of victims in cases of stalking. In this study, narcissistic individuals were more likely to dismiss harassing behaviour as non-stalking, while those with higher levels of psychopathy were less inclined to see the need for police intervention. Similarly, individuals with high levels of sadism were less likely to recognise the potential for causing distress or fear.^19^ Analogous results were found in relation to hands-off abuse, with sadism being the strongest predictor of online sexual victimisation, psychopathy for revenge porn propensity and narcissism for psychological intimate partner violence perpetration.^20^

Other studies have shown relationships between Machiavellianism, sadism, disinhibition, lack of perspective-taking and indulgent attitudes towards abuse.^21^ Looking at the broader sociocultural context, research^22^ also pointed to a link between acceptance of ideologies that affirm male supremacy over women (e.g. sexism, machismo and hypermasculinity), Dark Tetrad traits and tolerance of sexual violence.

In an attempt to deepen the understanding of the psychological pathways behind advocacy of violence against women, this study aimed to examine the relationship between Dark Tetrad personalities and vindictive rape (i.e. a vengeful and punitive response to perceived sexual transgressions) in non-incarcerated, community-based individuals. Specifically, the present study examined whether empathy could serve as a mediator between these study variables. Based on the conceptual framework and the literature review, the following hypotheses were proposed:

- H1: There is a significant relationship between Dark Tetrad traits, empathy and attitudes supportive of vindictive rape.
- H2: Attitudes supportive of vindictive rape are negatively correlated with empathy and positively correlated with Dark Tetrad traits.
- H3: Empathy is negatively correlated with Dark Tetrad traits.
- H4: Empathy mediates the relationship between Dark Tetrad traits and attitudes supportive of vindictive rape.

## Method

### Participants

Participants were 1548 individuals recruited from the general Italian population, including 1037 females (67.3%) and 503 males (32.7%). Most of them were of Italian nationality (*n* = 1530; 98.8%) and aged from 18 to 83 (*M*_age_ = 36.94; s.d._age_ = 15.72). Table 1 shows more details about participants’ sociodemographic characteristics.

**Table 1:** Sociodemographic characteristics

Sociodemographic characteristics	Sample (*n* = 1548)
Gender, *n* (%)^a^	
Male	503 (32.7)
Female	1037 (67.3)
Age, mean (s.d.)	36.94 (15.72)
Educational level, *n* (%)	
Primary school	5 (0.3)
Middle school	77 (5.0)
High school	671 (43.3)
Degree	665 (43.0)
Post-degree	130 (8.4)
Nationality, *n* (%)	
Italian	1530 (98.8)
Foreigner	18 (1.2)
Occupation, *n* (%)	
Employed	916 (59.2)
Unemployed	74 (4.8)
Student	463 (29.9)
Retired	95 (6.1)
Marital status, *n* (%)	
Single	486 (31.4)
In a relationship without cohabiting	447 (28.9)
Married or cohabiting	615 (39.7)

a. Preferred not to disclose in eight cases.

### Procedure

Data were collected from 1 July 2023 to 31 October 2023 through an anonymous online survey hosted on Google Forms. To reach a broader sociodemographic sample, we employed a snowball sampling strategy, starting by recruiting participants from major social networking sites and then encouraging them to share the survey link with their own networks. Exclusion criteria were defined, including age below 18 years, education level below 5 years, low Italian language proficiency and the presence of severe psychiatric and/or neurological disorders, which were determined by a yes/no self-report item. Only participants with complete data on all the outcome measures were retained. This process led to a final sample of 1548 individuals. All procedures contributing to this work comply with the ethical standards of the relevant national and institutional committees on human experimentation and with the Helsinki Declaration of 1975, as revised in 2013. All procedures involving human subjects were approved by the University of Turin Ethics Committee (protocol reference number 0289029). Participants were assured of data confidentiality and were informed that participation in the study was voluntary and unpaid. Written informed consent was obtained from all of them.

### Instruments

#### Sociodemographic and clinical information

Participants were asked to provide the following sociodemographic and clinical information: age, gender, educational level, nationality, occupation, marital status and the presence or history of psychiatric-neurological disorders (used as inclusion/exclusion criteria).

#### The Dark Triad Dirty Dozen (DTDD)

The Italian validated version of the DTDD^23,24^ is a self-report questionnaire comprising 12 items designed to measure the three Dark Triad traits. Participants were instructed to indicate their agreement on a scale ranging from 0 (Strongly disagree) to 4 (Strongly agree) with statements such as: ‘I tend to manipulate others to get my way’ (reflecting Machiavellianism), ‘I tend to lack remorse’ (indicating psychopathy) and ‘I tend to want others to admire me’ (representing narcissism).

The scale has demonstrated good or acceptable internal consistency in this study (Machiavellianism: *α* = 0.86; psychopathy: *α* = 0.70; narcissism: *α* = 0.82).

#### The Short Sadistic Impulse Scale (SSIS)

The Italian adaptation of the SSIS^25^ is a 10-item self-report scale aimed at evaluating sadistic tendencies at a subclinical level (i.e. indirect manifestations of sadism). The instrument was adapted into Italian following internationally accepted guidelines for cross-cultural scale translation. First, two independent bilingual researchers translated the original English items into Italian (forward translation). These two versions were compared and reconciled into a single provisional Italian version. Subsequently, a separate bilingual translator, blind to the original scale, back-translated the instrument into English (back-translation). The back-translation was then checked against the original items to ensure conceptual and semantic equivalence. Discrepancies were discussed and resolved by the translation team, and minor linguistic adjustments were made to preserve the meaning and tone of the original scale.

Participants rated the extent to which they agreed with each statement on a 5-point Likert-type scale ranging from 1 (Not at all like me) to 5 (Very like me). Examples of the SSIS items include ‘I have fantasies which involve hurting people’ and ‘I have humiliated others to keep them in line’. The sum of all the items’ ratings was computed as the final score, with higher scores reflecting greater sadistic inclinations. In the current research, the reliability was good (*α* = 0.80).

#### The Basic Empathy Scale (BES)

The Italian validated version of the BES^26,27^ comprises 20 items, each scored on a scale from 1 (Strongly disagree) to 5 (Strongly agree). The items are categorised into two factors: cognitive empathy and affective empathy. Nine items comprise the cognitive subscale, for which some examples are the following: ‘I find it hard to know when my friends are frightened’ and ‘I can often understand how people are feeling even before they tell me’. Meanwhile, the affective subscale, comprising 11 items, includes statements like ‘Seeing a person who has been angered has no impact on my feelings’ and ‘I don’t experience sadness when I witness others crying’. Total scores on the scale range from 20 (indicating a deficit in empathy) to 100 (reflecting a high level of empathy). In our sample, Cronbach’s α value was good for the BES total score (*α* = 0.86).

#### Vindictive Rape Attitude Questionnaire (VRAQ)

The Italian validated version of the VRAQ^28,29^ was used to assess the agreement to a form of retributive justice where the perpetrator punishes the female victim for perceived violations of sexual norms. Participants were asked to answer each of the 15 items (e.g. ‘If a woman cheats on her husband, I would have no sympathy if she gets raped.’) on a 5-point scale (with scores ranging from 1 = completely disagree to 5 = completely agree). Higher scores indicate stronger support of vindictive rape. In the current sample, the Cronbach’s α coefficient was 0.82.

### Data analysis

Statistical analyses were conducted using SPSS version 29.0 for Windows (IBM, Armonk, New York, USA). Normal distribution was assessed using the indices of asymmetry and kurtosis, which confirmed that all variables had a normal distribution. Descriptive data were calculated for the entire sample to obtain an overview of the sociodemographic and psychological characteristics of the participants. Descriptive data were presented as means with s.d. for continuous variables or as frequencies with percentages for categorical variables. Participants who failed to complete all study measures were automatically excluded from the data-set, resulting in a final sample with no missing data.

First, Pearson correlation analyses were conducted to assess the presence of significant associations between the target variables (e.g. Dark Tetrad personality traits, empathy and attitudes supportive of vindictive rape). Second, PROCESS macro 4 for SPSS (model type 4) was used to test the mediating role of empathy in the association between Dark Tetrad personality traits (as independent variables) and attitudes supportive of vindictive rape (as the dependent variable), controlling for the effect of gender.^30^ Only those variables with significant correlations with the dependent variable were included in the following mediation models. The bootstrap sample size was 5000, and if the value of zero was not within the 95% confidence interval, the mediating effect was considered significant.

## Results

### Correlation analyses

Bivariate correlations, score ranges, means and s.d. for the study variables are presented in Table 2.

**Table 2:** Bivariate correlations for the study variables

Variables	1	2	3	4	5	6
1. Machiavellianism	–					
2. Narcissism	0.57***	–				
3. Psychopathy	0.49***	0.32***	–			
4. Sadism	0.47***	0.32***	0.40***	–		
5. Empathy	−0.17***	0.02	−0.36***	−0.25***	–	
6. Vindictive rape	0.09***	0.05	0.08***	0.23***	−0.19***	–
7. Gender (0 = F; 1 = M)	0.19***	0.12***	0.24***	0.22***	−0.38***	0.16***
Range	0–16	0–16	0–16	10–47	37–100	15–75
Mean	2.99	5.55	3.96	15.09	76.49	25.17
s.d.	3.11	3.64	3.13	5.31	9.92	7.40

***p < .001.

### Mediation analyses

A set of three mediation models was employed to examine the effect of Dark Tetrad personality traits on attitudes supportive of vindictive rape as mediated by empathy, controlling for the gender of the participants.

#### Effects of Machiavellianism on attitudes supportive of vindictive rape through empathy

**Fig. 1:**
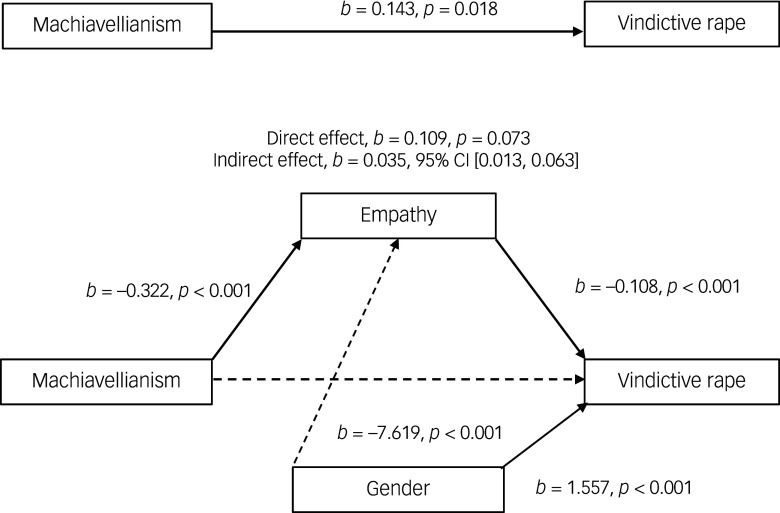
Model of Machiavellianism as a predictor of attitudes supportive of vindictive rape, mediated by empathy. The confidence interval for the indirect effect is a bias-corrected and accelerated bootstrapped confidence interval based on 5000 samples.

The first mediation analysis (Fig. 1) was conducted to examine whether the BES total score mediates the relationship between the Machiavellianism subscale of the DTDD and the VRAQ. The results indicated that there was no significant direct effect of Machiavellianism on VRAQ (*b* = 0.109, *β* = 0.046, *p* = 0.073). However, a significant indirect effect of Machiavellianism on VRAQ through the BES total score was observed (*b* = 0.035, *β* = 0.015, bias-corrected and accelerated (BCA) CI (0.013, 0.063)). This suggests that higher levels of Machiavellianism are associated with stronger support for vindictive rape, indirectly through lower levels of empathy. The indirect pathway accounted for approximately 37% of the total explained variance in the model.

#### Effects of psychopathy on attitudes supportive of vindictive rape through empathy

**Fig. 2:**
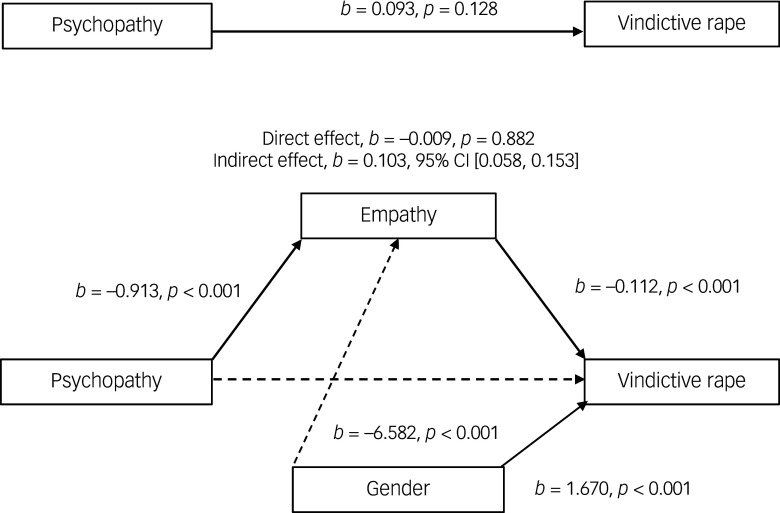
Model of psychopathy as a predictor of attitudes supportive of vindictive rape, mediated by empathy. The confidence interval for the indirect effect is a bias-corrected and accelerated bootstrapped confidence interval based on 5000 samples.

The second mediation analysis (Fig. 2) was conducted to examine the effect of the BES total score in mediating the association between the psychopathy subscale of the DTDD and the VRAQ. Results showed a significant indirect effect of psychopathy on the VRAQ through the BES total score (*b* = 0.103, *β* = 0.044, BCa CI (0.058, 0.153). In contrast, no evidence was found that psychopathy had a direct effect on attitudes supporting vindictive rape (*b* = −0.009, *β* = −0.004, *p* = 0.882). The indirect pathway explained approximately 39% of the variance attributable to the model.

#### Effects of sadism on attitudes supportive of vindictive rape through empathy

**Fig. 3:**
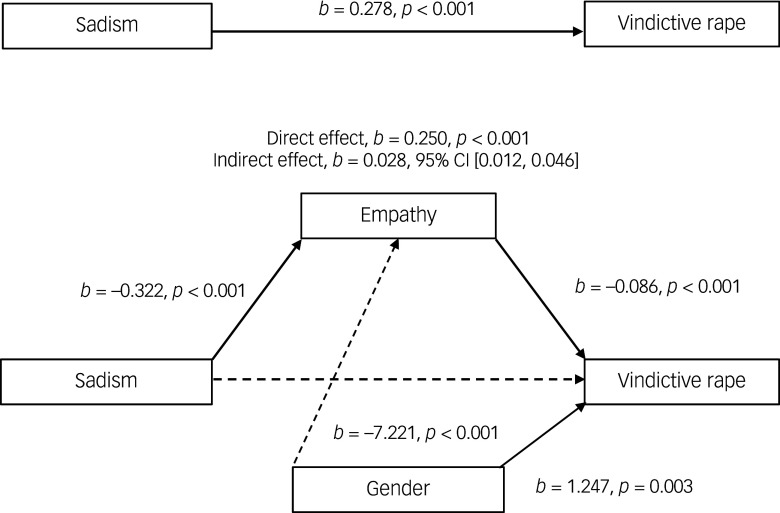
Model of sadism as a predictor of attitudes supportive of vindictive rape, mediated by empathy. The confidence interval for the indirect effect is a bias-corrected and accelerated bootstrapped confidence interval based on 5000 samples.

In the third mediation model (Fig. 3), sadism was significantly directly related to attitudes supporting vindictive rape (*b* = 0.250, *β* = 0.180, *p* < 0.001). When assessing the indirect path, the positive association between sadistic traits and support for vindictive rape through empathy (*b* = 0.028, *β* = 0.020, BCa CI = (0.012–0.046)) was significant. The indirect pathway accounted for approximately 15% of the total explained variance in the model.

## Discussion

The main aim of the present study was to examine whether empathy mediates the association between Dark Tetrad personality traits and attitudes endorsing vindictive rape in a non-criminal, community-based sample, with gender controlled for. Consistent with our hypotheses, we found that lower empathy partially accounted for the link between Dark traits and rape-supportive attitudes, particularly for Machiavellianism, psychopathy and sadism, whereas narcissism did not show a significant association. The findings support previous studies that have linked Dark Tetrad personality traits to a variety of socially displeasing or even criminal behaviours, ranging from animal cruelty^31^ to cyberbullying.^32^

Within this framework, the current study revealed associations between Dark Tetrad personalities, diminished empathy and support for retributive justice in response to perceived violations of traditional sexual norms by women. Specifically, we found an indirect relationship between Machiavellianism and attitudes supportive of vindictive rape. That is, personality traits related to ruthlessness, manipulativeness and opportunism appear to characterise those individuals who tend to be more tolerant of rape as a form of revenge against women, and this association is mediated by reduced empathy. These findings are consistent with previous work on mechanisms of moral disengagement that explain both hostile and benevolent sexism associated with Machiavellianism among community men.^33^ It is important to note, however, that the effect sizes observed for Machiavellianism were small, suggesting that while the associations are statistically significant, they represent subtle tendencies that may accumulate or interact with contextual factors rather than strong individual-level effects. From a broader perspective, these subtle but systematic tendencies may contribute to a cultural climate where grievance-driven violence is implicitly tolerated, highlighting the need for preventive social policies aimed at reducing acceptance towards gender-based aggression and challenging retributive narratives in public discourse. In this regard, efforts to address patriarchal and heteronormative scripts through education and media literacy may represent an important complement to individual-focused interventions.

The findings also showed that psychopathy was indirectly (but not directly) related to attitudes that support vindictive rape. This result is somewhat in line with previous research that has found a relationship between psychopathy and intimate partner violence.^34^ In the present study, the association between psychopathy and vindictive rape was explained by empathy: shallow affect, lack of concern for the feelings or well-being of others and a diminished sense of guilt and remorse are indeed recognised as central features of psychopathy.^35^ These two findings are particularly relevant, as Machiavellianism and psychopathy have an effect in fuelling thoughts of revenge against the partner in intimate relationships.^36^ Consistent with this explanation, the effect sizes for psychopathy were also modest, reinforcing the notion that personality-related risk factors may exert their influence through incremental and cumulative patterns rather than through large, direct effects. These results underscore the potential value of public-facing interventions that promote empathic concern and emotional literacy, as well as policy measures supporting early-prevention programmes in schools and digital spaces, particularly targeting adolescents and young adults where antisocial cognitive patterns may first consolidate. Such findings also suggest that prevention and treatment programmes targeting violent and coercive behaviours may benefit from including modules focused on emotional responsiveness and perspective-taking.

In addition, there was evidence of a direct and indirect relationship between sadistic traits and attitudes that support vindictive rape. This finding aligns with earlier research that demonstrated a positive impact of everyday sadism, particularly of a physical nature, on both sexual aggression commission and the lack of ability to recognise it as such.^37^ Although sadism showed comparatively larger standardised coefficients than Machiavellianism and psychopathy, the effects still fall within a small range. Thus, while highly relevant theoretically, they should be interpreted within the broader context of multifactorial contributors to sexual aggression–related attitudes. Policy initiatives focusing solely on punitive approaches to sexual violence may therefore be insufficient: prevention frameworks should also consider motivational and affective processes associated with sadism and include targeted psychoeducational programmes addressing harmful pleasure-seeking dynamics in aggression.

The current study reflects the role of empathy as a mediator in the interaction between Dark traits and attitudes that support vindictive rape and confirms the protective role of the ability to understand and share the emotional experiences of others from aversive patterns of psychological functioning and antisocial tendencies.^16^ This supports public-health models advocating for empathy-training and socio-emotional skill-building in educational settings as protective factors against misogynistic ideologies and rape-supportive beliefs, and suggests that community-based interventions may have broad preventive value. Beyond its mediating function, empathy may also act as an active inhibitory mechanism that restrains punitive and vengeful impulses by fostering emotional attunement, perspective-taking and recognition of victims’ suffering. Individuals with stronger empathic abilities may therefore be less inclined to morally disengage or dehumanise women perceived as violating sexual norms, reducing the psychological distance that facilitates blame-driven aggression. As with the work of Pina and colleagues,^38^ in which narcissism was examined in relation to willingness to engage in image-based sexual abuse, we found no association between narcissism and attitudes that support vindictive rape. Conversely, previous research by Blinkhorn and colleagues^39^ found that individuals with higher narcissism tended to be more accepting of various types of violence (e.g. war, corporal punishment of children, institutional violence and intimate partner violence), whereas in another study^40^ women who exhibited higher levels of narcissism were more likely to self-report committing sexually coercive acts, which may be found within the different sub-facets of narcissism, and therefore, this finding requires future investigation. Moreover, recent evidence suggests that Dark personality traits are intertwined with relational functioning and attachment dynamics, with those higher in such traits reporting distinct attitudes towards romantic relationships^41^ and demonstrating maladaptive socio-emotional profiles characterised by reduced emotional awareness and empathic concern.^42^ In this sense, future research should also examine whether cognitive and affective components of empathy differentially relate to rape-supportive attitudes among individuals high in Dark personality traits. Taken together, these results reaffirm our hypothesis that empathy may operate as a protective factor against rape-supportive attitudes, particularly among individuals scoring higher in certain Dark traits. Findings from such work could inform policy development, risk-screening practices and offender-rehabilitation strategies, as well as guide evidence-based educational initiatives and media campaigns promoting expectations of respectful relationships and gender equality.

### Limitations

These findings offer a new perspective on the relationship between personality traits and violence against women, focusing on attitudes towards women’s sexual roles and rape as a means of asserting dominance, humiliation and attempted retaliation. Nevertheless, the results should be interpreted with caution, taking into account the specific limitations within the research characteristics. The use of self-report scales may have introduced bias due to participants’ potential lack of self-awareness and social desirability effects. This could have contributed to the relatively low overall level of agreement with the VRAQ items. Moreover, the sample was characterised by a gender imbalance, with a predominance of female participants, which should be considered when interpreting the observed associations. It is also essential to recognise the constraints of mediation analyses conducted in cross-sectional, self-report designs, which cannot establish causality and only allow for the identification of associations.

In conclusion, to our knowledge, the present study represents the first contribution to understanding the mediating effect of empathy in the relationship between Dark Tetrad personalities and attitudes that support vindictive rape in the general population. In doing so, it extends the literature on how personality traits shape perceptions and attitudes towards violence against women, focusing on sexual violence. This aligns with growing evidence indicating that Dark personality traits are linked to emotional dysregulation and compromised empathic capacities,^42^ as well as hostile and exploitative interpersonal orientations within intimate contexts.^41^ This is of importance given the prevalence of this phenomenon, as the World Health Organization’s global estimates^43^ suggest that approximately one in three women worldwide have experienced either physical and/or sexual intimate partner violence or non-partner sexual violence at some point in their lives.

Furthermore, the public response to cases of sexual violence plays a role in shaping the social climate surrounding this issue, potentially perpetuating misconceptions and the attribution of responsibility to victims.^44^ The latter has important implications for jury decision-making^45^ and, more broadly, the perpetuation of gender system justification and existing social inequalities for women.

## Data Availability

The data that support the findings of this study are available from the corresponding author, M.D.T., upon reasonable request.
